# Supplementary material for: A Traffic Light System to Maximize Carbohydrate Cryoprotectants’ Effectivity in Nanostructured Lipid Carriers’ Lyophilization
Source: Pharmaceutics. 2021 Aug 25;13(9):1330. doi: 10.3390/pharmaceutics13091330 (PMC8470209; doi:10.3390/pharmaceutics13091330)
Supplement: Supplementary file 1 [file pharmaceutics-13-01330-s001.zip › pharmaceutics-1320252-supplementary.pdf]

pharmaceutics

Supplementary Materials: A Traffic Light System to Maximize Carbohydrate Cryoprotectants' Effectivity in Nanostructured Lipid Carriers' Lyophilization

Table S1. Set of combined IF-THEN rules for Δ size of the first NFL model. Degree of membership in parentheses.

Rules for Δ size		
1. IF Velocity is Fast AND CP is Fructose AND IF %CP is HIGH AND CP is Fructose	THEN Δ size (nm) is	HIGH (0.60)
2. IF Velocity is Fast AND CP is Fructose AND IF %CP is LOW AND CP is Fructose	THEN Δ size (nm) is	LOW (0.82)
3. IF Velocity is Fast AND CP is Fructose AND IF %CP is MID AND CP is Fructose	THEN Δ size (nm) is	LOW (0.89)
4. IF Velocity is Slow AND CP is Fructose AND IF %CP is HIGH AND CP is Fructose	THEN Δ size (nm) is	HIGH (0.86)
5. IF Velocity is Slow AND CP is Fructose AND IF %CP is LOW AND CP is Fructose	THEN Δ size (nm) is	LOW (0.56)
6. IF Velocity is Slow AND CP is Fructose AND IF %CP is MID AND CP is Fructose	THEN Δ size (nm) is	LOW (0.63)
7. IF Velocity is Fast AND CP is Glucose AND IF %CP is HIGH AND CP is Glucose	THEN Δ size (nm) is	LOW (0.56)
8. IF Velocity is Fast AND CP is Glucose AND IF %CP is LOW AND CP is Glucose	THEN Δ size (nm) is	LOW (0.99)
9. IF Velocity is Fast AND CP is Glucose AND IF %CP is MID AND CP is Glucose	THEN Δ size (nm) is	LOW (0.90)
10. IF Velocity is Slow AND CP is Glucose AND IF %CP is HIGH AND CP is Glucose	THEN Δ size (nm) is	HIGH (0.72)
11. IF Velocity is Slow AND CP is Glucose AND IF %CP is LOW AND CP is Glucose	THEN Δ size (nm) is	LOW (0.72)
12. IF Velocity is Slow AND CP is Glucose AND IF %CP is MID AND CP is Glucose	THEN Δ size (nm) is	LOW (0.62)
13. IF Velocity is Fast AND CP is Lactose AND IF %CP is HIGH AND CP is Lactose	THEN Δ size (nm) is	LOW (0.74)

14. IF Velocity is Fast AND CP is Lactose AND IF %CP is LOW AND CP is Lactose	THEN Δ size (nm) is	LOW (0.81)
15. IF Velocity is Fast AND CP is Lactose AND IF %CP is MID AND CP is Lactose	THEN Δ size (nm) is	LOW (0.88)
16. IF Velocity is Slow AND CP is Lactose AND IF %CP is HIGH AND CP is Lactose	THEN Δ size (nm) is	LOW (0.66)
17. IF Velocity is Slow AND CP is Lactose AND IF %CP is LOW AND CP is Lactose	THEN Δ size (nm) is	LOW (0.73)
18. IF Velocity is Slow AND CP is Lactose AND IF %CP is MID AND CP is Lactose	THEN Δ size (nm) is	LOW (0.81)
19. IF Velocity is Fast AND CP is Mannitol AND IF %CP is HIGH AND CP is Mannitol	THEN Δ size (nm) is	LOW (0.71)
20. IF Velocity is Fast AND CP is Mannitol AND IF %CP is LOW AND CP is Mannitol	THEN Δ size (nm) is	HIGH (0.77)
21. IF Velocity is Fast AND CP is Mannitol AND IF %CP is MID AND CP is Mannitol	THEN Δ size (nm) is	LOW (0.60)
22. IF Velocity is Slow AND CP is Mannitol AND IF %CP is HIGH AND CP is Mannitol	THEN Δ size (nm) is	LOW (0.62)
23. IF Velocity is Slow AND CP is Mannitol AND IF %CP is LOW AND CP is Mannitol	THEN Δ size (nm) is	HIGH (0.86)
24. IF Velocity is Slow AND CP is Mannitol AND IF %CP is MID AND CP is Mannitol	THEN Δ size (nm) is	LOW (0.50)
25. IF Velocity is Fast AND CP is Sorbitol AND IF %CP is HIGH AND CP is Sorbitol	THEN Δ size (nm) is	HIGH (0.50)
26. IF Velocity is Fast AND CP is Sorbitol AND IF %CP is LOW AND CP is Sorbitol	THEN Δ size (nm) is	LOW (0.51)
27. IF Velocity is Fast AND CP is Sorbitol AND IF %CP is MID AND CP is Sorbitol	THEN Δ size (nm) is	LOW (0.66)
28. IF Velocity is Slow AND CP is Sorbitol AND IF %CP is HIGH AND CP is Sorbitol	THEN Δ size (nm) is	HIGH (0.61)
29. IF Velocity is Slow AND CP is Sorbitol AND IF %CP is LOW AND CP is Sorbitol	THEN Δ size (nm) is	HIGH (0.60)

30. IF Velocity is Slow AND CP is Sorbitol AND IF %CP is MID AND CP is Sorbitol THEN	THEN Δ size (nm) is	LOW (0.55)
31. IF Velocity is Fast AND CP is Sucrose AND IF %CP is HIGH AND CP is Sucrose	THEN Δ size (nm) is	LOW (0.94)
32. IF Velocity is Fast AND CP is Sucrose AND IF %CP is LOW AND CP is Sucrose	THEN Δ size (nm) is	HIGH (0.75)
33. IF Velocity is Fast AND CP is Sucrose AND IF %CP is MID AND CP is Sucrose	THEN Δ size (nm) is	LOW (0.76)
34. IF Velocity is Slow AND CP is Sucrose AND IF %CP is HIGH AND CP is Sucrose	THEN Δ size (nm) is	LOW (0.88)
35. IF Velocity is Slow AND CP is Sucrose AND IF %CP is LOW AND CP is Sucrose	THEN Δ size (nm) is	HIGH (0.82)
36. IF Velocity is Slow AND CP is Sucrose AND IF %CP is MID AND CP is Sucrose	THEN Δ size (nm) is	LOW (0.70)
37. IF Velocity is Fast AND CP is Trehalose AND IF %CP is HIGH AND CP is Trehalose	THEN Δ size (nm) is	LOW (0.71)
38. IF Velocity is Fast AND CP is Trehalose AND IF %CP is LOW AND CP is Trehalose	THEN Δ size (nm) is	LOW (0.68)
39. IF Velocity is Fast AND CP is Trehalose AND IF %CP is MID AND CP is Trehalose	THEN Δ size (nm) is	LOW (0.85)
40. IF Velocity is Slow AND CP is Trehalose AND IF %CP is HIGH AND CP is Trehalose	THEN Δ size (nm) is	LOW (0.72)
41. IF Velocity is Slow AND CP is Trehalose AND IF %CP is LOW AND CP is Trehalose	THEN Δ size (nm) is	LOW (0.69)
42. IF Velocity is Slow AND CP is Trehalose AND IF %CP is MID AND CP is Trehalose	THEN Δ size (nm) is	LOW (0.86)

Table S2. Set of combined IF-THEN rules for Δ PdI of the first NFL model. Degree of membership in parentheses.

**Rules for Δ PdI**		
1. IF %CP is LOW AND CP is Fructose AND IF Velocity is Fast AND IF %CP is MID-3	THEN Δ PdI is	LOW (1.00)
2. IF %CP is LOW AND CP is Fructose AND IF Velocity is Fast AND IF % CP is LOW-1	THEN Δ PdI is	LOW (1.00)

3. IF %CP is LOW AND CP is Fructose AND IF Velocity is Fast AND IF %CP is MID-2	THEN Δ PdI is	LOW (0.92)
4. IF %CP is HIGH AND CP is Fructose AND IF Velocity is Fast AND IF %CP is MID-3	THEN Δ PdI is	LOW (0.71)
5. IF %CP is HIGH AND CP is Fructose AND IF Velocity is Fast AND IF %CP is HIGH-4	THEN Δ PdI is	HIGH (0.63)
6. IF %CP is LOW AND CP is Fructose AND IF Velocity is Slow AND IF %CP is MID-3	THEN Δ PdI is	LOW (0.88)
7. IF %CP is LOW AND CP is Fructose AND IF Velocity is Slow AND IF %CP is LOW-1	THEN Δ PdI is	LOW (0.87)
8. IF %CP is LOW AND CP is Fructose AND IF Velocity is Slow AND IF %CP is MID-2	THEN Δ PdI is	LOW (0.77)
9. IF % CP is HIGH AND CP is Fructose AND IF Velocity is Slow AND IF %CP is MID-3	THEN Δ PdI is	LOW (0.56)
10. IF %CP is HIGH AND CP is Fructose AND IF Velocity is Slow AND IF %CP is HIGH-4	THEN Δ PdI is	HIGH (0.78)
11. IF %CP is LOW AND CP is Glucose AND IF Velocity is Fast AND IF %CP is MID-3	THEN Δ PdI is	LOW (1.00)
12. IF %CP is LOW AND CP is Glucose AND IF Velocity is Fast AND IF %CP is LOW-1	THEN Δ PdI is	LOW (1.00)
13. IF %CP is LOW AND CP is Glucose AND IF Velocity is Fast AND IF %CP is MID-2	THEN Δ PdI is	LOW (0.97)
14. IF %CP is HIGH AND CP is Glucose AND IF Velocity is Fast AND IF %CP is MID-3	THEN Δ PdI is	LOW (0.62)
15. IF %CP is HIGH AND CP is Glucose AND IF Velocity is Fast AND IF %CP is HIGH-4	THEN Δ PdI is	HIGH (0.72)
16. IF %CP is LOW AND CP is Glucose AND IF Velocity is Slow AND IF %CP is MID-3	THEN Δ PdI is	LOW (0.94)
17. IF %CP is LOW AND CP is Glucose AND IF Velocity is Slow AND IF %CP is LOW-1	THEN Δ PdI is	LOW (0.92)
18. IF %CP is LOW AND CP is Glucose AND IF Velocity is Slow AND IF %CP is MID-2	THEN Δ PdI is	LOW (0.82)

19. IF %CP is HIGH AND CP is Glucose AND IF Velocity is Slow AND IF %CP is MID-3	THEN Δ PdI is	HIGH (0.54)
20. IF %CP is HIGH AND CP is Glucose AND IF Velocity is Slow AND IF %CP is HIGH-4	THEN Δ PdI is	HIGH (0.87)
21. IF %CP is LOW AND CP is Lactose AND IF Velocity is Fast AND IF %CP is MID-3	THEN Δ PdI is	LOW (1.00)
22. IF %CP is LOW AND CP is Lactose AND IF Velocity is Fast AND IF %CP is LOW-1	THEN Δ PdI is	LOW (1.00)
23. IF %CP is LOW AND CP is Lactose AND IF Velocity is Fast AND IF %CP is MID-2	THEN Δ PdI is	LOW (0.90)
24. IF %CP is HIGH AND CP is Lactose AND IF Velocity is Fast AND IF %CP is MID-3	THEN Δ PdI is	LOW (0.73)
25. IF %CP is HIGH AND CP is Lactose AND IF Velocity is Fast AND IF %CP is HIGH-4	THEN Δ PdI is	HIGH (0.61)
26. IF %CP is LOW AND CP is Lactose AND IF Velocity is Slow AND IF %CP is MID-3	THEN Δ PdI is	LOW (0.86)
27. IF %CP is LOW AND CP is Lactose AND IF Velocity is Slow AND IF %CP is LOW-1	THEN Δ PdI is	LOW (0.85)
28. IF %CP is LOW AND CP is Lactose AND IF Velocity is Slow AND IF %CP is MID-2	THEN Δ PdI is	LOW (0.75)
29. IF %CP is HIGH AND CP is Lactose AND IF Velocity is Slow AND IF %CP is MID-3	THEN Δ PdI is	LOW (0.58)
30. IF %CP is HIGH AND CP is Lactose AND IF Velocity is Slow AND IF %CP is HIGH-4	THEN Δ PdI is	HIGH (0.76)
31. IF %CP is HIGH AND CP is Mannitol AND IF Velocity is Fast AND IF %CP is MID-3	THEN Δ PdI is	LOW (1.00)
32. IF %CP is LOW AND CP is Mannitol AND IF Velocity is Fast AND IF %CP is MID-3	THEN Δ PdI is	LOW (0.78)
33. IF %CP is LOW AND CP is Mannitol AND IF Velocity is Fast AND IF %CP is LOW-1	THEN Δ PdI is	LOW (0.77)
34. IF %CP is HIGH AND CP is Mannitol AND IF Velocity is Fast AND IF %CP is HIGH-4	THEN Δ PdI is	LOW (0.72)

35. IF %CP is LOW AND CP is Mannitol AND IF Velocity is Fast AND IF %CP is MID-2	THEN Δ PdI is	LOW (0.67)
36. IF %CP is HIGH AND CP is Mannitol AND IF Velocity is Slow AND IF %CP is MID-3	THEN Δ PdI is	LOW (0.91)
37. IF %CP is LOW AND CP is Mannitol AND IF Velocity is Slow AND IF %CP is MID-3	THEN Δ PdI is	LOW (0.63)
38. IF %CP is LOW AND CP is Mannitol AND IF Velocity is Slow AND IF %CP is LOW-1	THEN Δ PdI is	LOW (0.61)
39. IF %CP is HIGH AND CP is Mannitol AND IF Velocity is Slow AND IF %CP is HIGH-4	THEN Δ PdI is	LOW (0.57)
40. IF %CP is LOW AND CP is Mannitol AND IF Velocity is Slow AND IF %CP is MID-2	THEN Δ PdI is	LOW (0.52)
41. IF %CP is HIGH AND CP is Sorbitol AND IF Velocity is Fast AND IF %CP is MID-3	THEN Δ PdI is	LOW (0.88)
42. IF %CP is LOW AND CP is Sorbitol AND IF Velocity is Fast AND IF %CP is MID-3	THEN Δ PdI is	LOW (0.83)
43. IF %CP is LOW AND CP is Sorbitol AND IF Velocity is Fast AND IF %CP is LOW-1	THEN Δ PdI is	LOW (0.82)
44. IF %CP is LOW AND CP is Sorbitol AND IF Velocity is Fast AND IF %CP is MID-2	THEN Δ PdI is	LOW (0.72)
45. IF %CP is HIGH AND CP is Sorbitol AND IF Velocity is Fast AND IF %CP is HIGH-4	THEN Δ PdI is	LOW (0.54)
46. IF %CP is HIGH AND CP is Sorbitol AND IF Velocity is Slow AND IF %CP is MID-3	THEN Δ PdI is	LOW (0.72)
47. IF %CP is LOW AND CP is Sorbitol AND IF Velocity is Slow AND IF %CP is MID-3	THEN Δ PdI is	LOW (0.68)
48. IF %CP is LOW AND CP is Sorbitol AND IF Velocity is Slow AND IF %CP is LOW-1	THEN Δ PdI is	LOW (0.67)
49. IF %CP is LOW AND CP is Sorbitol AND IF Velocity is Slow AND IF %CP is MID-2	THEN Δ PdI is	LOW (0.57)
50. IF %CP is HIGH AND CP is Sorbitol AND IF Velocity is Slow AND IF %CP is HIGH-4	THEN Δ PdI is	HIGH (0.61)

51. IF %CP is HIGH AND CP is Sucrose AND IF Velocity is Fast AND IF %CP is MID-3	THEN Δ PdI is	LOW (1.00)
52. IF %CP is HIGH AND CP is Sucrose AND IF Velocity is Fast AND IF %CP is HIGH-4	THEN Δ PdI is	LOW (0.85)
53. IF %CP is LOW AND CP is Sucrose AND IF Velocity is Fast AND IF %CP is MID-3	THEN Δ PdI is	LOW (0.77)
54. IF %CP is LOW AND CP is Sucrose AND IF Velocity is Fast AND IF %CP is LOW-1	THEN Δ PdI is	LOW (0.75)
55. IF %CP is LOW AND CP is Sucrose AND IF Velocity is Fast AND IF %CP is MID-2	THEN Δ PdI is	LOW (0.65)
56. IF %CP is HIGH AND CP is Sucrose AND IF Velocity is Slow AND IF %CP is MID-3	THEN Δ PdI is	LOW (1.00)
57. IF %CP is HIGH AND CP is Sucrose AND IF Velocity is Slow AND IF %CP is LOW-1	THEN Δ PdI is	LOW (1.00)
58. IF %CP is HIGH AND CP is Sucrose AND IF Velocity is Slow AND IF %CP is HIGH-4	THEN Δ PdI is	LOW (0.70)
59. IF %CP is LOW AND CP is Sucrose AND IF Velocity is Slow AND IF %CP is MID-3	THEN Δ PdI is	LOW (0.61)
60. IF %CP is LOW AND CP is Sucrose AND IF Velocity is Slow AND IF %CP is LOW-1	THEN Δ PdI is	LOW (0.60)
61. IF %CP is LOW AND CP is Sucrose AND IF Velocity is Slow AND IF %CP is MID-2	THEN Δ PdI is	LOW (0.50)
62. IF %CP is HIGH AND CP is Trehalose AND IF Velocity is Fast AND IF %CP is MID-3	THEN Δ PdI is	LOW (1.00)
63. IF %CP is LOW AND CP is Trehalose AND IF Velocity is Fast AND IF %CP is MID-3	THEN Δ PdI is	LOW (0.96)
64. IF %CP is LOW AND CP is Trehalose AND IF Velocity is Fast AND IF %CP is LOW-1	THEN Δ PdI is	LOW (0.94)
65. IF %CP is LOW AND CP is Trehalose AND IF Velocity is Fast AND IF %CP is MID-2	THEN Δ PdI is	LOW (0.84)
66. IF %CP is HIGH AND CP is Trehalose AND IF Velocity is Fast AND IF %CP is HIGH-4	THEN Δ PdI is	LOW (0.69)

67. IF %CP is HIGH AND CP is Trehalose AND IF Velocity is Slow AND IF %CP is MID-3	THEN Δ PdI is	LOW (0.88)
68. IF %CP is LOW AND CP is Trehalose AND IF Velocity is Slow AND IF %CP is MID-3	THEN Δ PdI is	LOW (0.81)
69. IF %CP is LOW AND CP is Trehalose AND IF Velocity is Slow AND IF %CP is LOW-1	THEN Δ PdI is	LOW (0.79)
70. IF %CP is LOW AND CP is Trehalose AND IF Velocity is Slow AND IF %CP is MID-2	THEN Δ PdI is	LOW (0.69)
71. IF %CP is HIGH AND CP is Trehalose AND IF Velocity is Slow AND IF %CP is HIGH-4	THEN Δ PdI is	LOW (0.54)

Table S3. Set of combined IF-THEN rules for Δ ZP of the first NFL model. Degree of membership in parentheses.

**Rules for Δ ZP**		
1. IF %CP is LOW AND IF CP is Fructose AND Velocity is Fast	THEN Δ ZP is	LOW (0.51)
2. IF %CP is HIGH AND IF CP is Fructose AND Velocity is Fast	THEN Δ ZP is	HIGH (0.67)
3. IF %CP is LOW AND IF CP is Fructose AND Velocity is Slow	THEN Δ ZP is	LOW (0.54)
4. IF %CP is HIGH AND IF CP is Fructose AND Velocity is Slow	THEN Δ ZP is	HIGH (0.64)
5. IF %CP is LOW AND IF CP is Glucose AND Velocity is Fast	THEN Δ ZP is	LOW (0.99)
6. IF %CP is HIGH AND IF CP is Glucose AND Velocity is Fast	THEN Δ ZP is	LOW (0.81)
7. IF %CP is LOW AND IF CP is Glucose AND Velocity is Slow	THEN Δ ZP is	LOW (0.71)
8. IF %CP is HIGH AND IF CP is Glucose AND Velocity is Slow	THEN Δ ZP is	LOW (0.54)
9. IF %CP is LOW AND IF CP is Lactose AND Velocity is Fast	THEN Δ ZP is	HIGH (0.56)
10. IF %CP is HIGH AND IF CP is Lactose AND Velocity is Fast	THEN Δ ZP is	HIGH (0.74)

11. IF %CP is LOW AND IF CP is Lactose AND Velocity is Slow	THEN Δ ZP is	LOW (0.71)
12. IF %CP is HIGH AND IF CP is Lactose AND Velocity is Slow	THEN Δ ZP is	LOW (0.54)
13. IF %CP is LOW AND IF CP is Mannitol AND Velocity is Fast	THEN Δ ZP is	LOW (0.63)
14. IF %CP is HIGH AND IF CP is Mannitol AND Velocity is Fast	THEN Δ ZP is	HIGH (0.55)
15. IF %CP is LOW AND IF CP is Mannitol AND Velocity is Slow	THEN Δ ZP is	LOW (0.83)
16. IF %CP is HIGH AND IF CP is Mannitol AND Velocity is Slow	THEN Δ ZP is	LOW (0.66)
17. IF %CP is LOW AND IF CP is Sorbitol AND Velocity is Fast	THEN Δ ZP is	LOW (0.83)
18. IF %CP is HIGH AND IF CP is Sorbitol AND Velocity is Fast	THEN Δ ZP is	LOW (0.66)
19. IF %CP is LOW AND IF CP is Sorbitol AND Velocity is Slow	THEN Δ ZP is	LOW (0.80)
20. IF %CP is HIGH AND IF CP is Sorbitol AND Velocity is Slow	THEN Δ ZP is	LOW (0.62)
21. IF %CP is LOW AND IF CP is Sucrose AND Velocity is Fast	THEN Δ ZP is	LOW (0.77)
22. IF %CP is HIGH AND IF CP is Sucrose AND Velocity is Fast	THEN Δ ZP is	LOW (0.59)
23. IF %CP is LOW AND IF CP is Sucrose AND Velocity is Slow	THEN Δ ZP is	LOW (0.73)
24. IF %CP is HIGH AND IF CP is Sucrose AND Velocity is Slow	THEN Δ ZP is	LOW (0.55)
25. IF %CP is LOW AND IF CP is Trehalose AND Velocity is Fast	THEN Δ ZP is	LOW (0.55)
26. IF %CP is HIGH AND IF CP is Trehalose AND Velocity is Fast	THEN Δ ZP is	HIGH (0.63)

27. IF %CP is LOW AND IF CP is Trehalose AND Velocity is Slow	THEN Δ ZP is	LOW (0.81)
28. IF %CP is HIGH AND IF CP is Trehalose AND Velocity is Slow	THEN Δ ZP is	LOW (0.64)

Table S4. Set of IF-THEN rules for Δ size of the second NFL model. Degree of membership in parentheses.

**Rules for Δ size**		
Submodel 1		
1. IF Osmolarity is LOW AND MW is LOW	THEN Δ size (nm) is	LOW (1.00)
2. IF Osmolarity is LOW AND MW is MID	THEN Δ size (nm) is	HIGH (1.00)
3. IF Osmolarity is LOW AND MW is HIGH	THEN Δ size (nm) is	LOW (0.64)
4. IF Osmolarity is MID AND MW is LOW	THEN Δ size (nm) is	LOW (0.77)
5. IF Osmolarity is MID AND MW is MID	THEN Δ size (nm) is	LOW (1.00)
6. IF Osmolarity is MID AND MW is HIGH	THEN Δ size (nm) is	LOW (1.00)
7. IF Osmolarity is HIGH AND MW is LOW	THEN Δ size (nm) is	HIGH (0.87)
8. IF Osmolarity is HIGH AND MW is MID	THEN Δ size (nm) is	LOW (1.00)
9. IF Osmolarity is HIGH AND MW is HIGH	THEN Δ size (nm) is	LOW (1.00)
Submodel 2		
10. IF Speed is Fast AND MW is LOW	THEN Δ size (nm) is	LOW (0.77)
11. IF Speed is Fast AND MW is MID	THEN Δ size (nm) is	HIGH (1.00)
12. IF Speed is Fast AND MW is HIGH	THEN Δ size (nm) is	LOW (0.89)
13. IF Speed is Slow AND MW is LOW	THEN Δ size (nm) is	HIGH (0.77)
14. IF Speed is Slow AND MW is MID	THEN Δ size (nm) is	LOW (1.00)
15. IF Speed is Slow AND MW is HIGH	THEN Δ size (nm) is	LOW (0.81)

It should be noted that the combination of inputs that led to the highest value of the output are highlighted in blue, while the combination of inputs giving the lowest value are highlighted in red.

Table S5. Set of IF-THEN rules for Δ PdI of the second NFL model. Degree of membership in parentheses.

**Rules for Δ PdI**		
Submodel 1		
1. IF Osmolarity is LOW AND MW is LOW	THEN Δ PdI is	LOW (0.67)
2. IF Osmolarity is LOW AND MW is MID	THEN Δ PdI is	HIGH (1.00)
3. IF Osmolarity is LOW AND MW is HIGH	THEN Δ PdI is	HIGH (0.51)
4. IF Osmolarity is MID AND MW is LOW	THEN Δ PdI is	HIGH (0.69)
5. IF Osmolarity is MID AND MW is MID	THEN Δ PdI is	LOW (1.00)
6. IF Osmolarity is MID AND MW is HIGH	THEN Δ PdI is	HIGH (0.65)
7. IF Osmolarity is HIGH AND MW is LOW	THEN Δ PdI is	HIGH (1.00)
8. IF Osmolarity is HIGH AND MW is MID	THEN Δ PdI is	LOW (1.00)
9. IF Osmolarity is HIGH AND MW is HIGH	THEN Δ PdI is	LOW (1.00)
Submodel 2		
10. IF Speed is Fast	THEN Δ PdI is	LOW (1.00)
11. IF Speed is Slow	THEN Δ PdI is	LOW (0.93)

It should be noted that the combination of inputs that led to the highest value of the output are highlighted in blue, while the combination of inputs giving the lowest value are highlighted in red.

Table S6. Set of IF-THEN rules for Δ ZP of the second NFL model. Degree of membership in parentheses.

**Rules for Δ ZP**		
Submodel 1		
1. IF Osmolarity is LOW	THEN Δ ZP is	LOW (0.65)
2. IF Osmolarity is HIGH	THEN Δ ZP is	LOW (0.57)

It should be noted that the combination of inputs that led to the highest value of the output are highlighted in blue, while the combination of inputs giving the lowest value are highlighted in red.

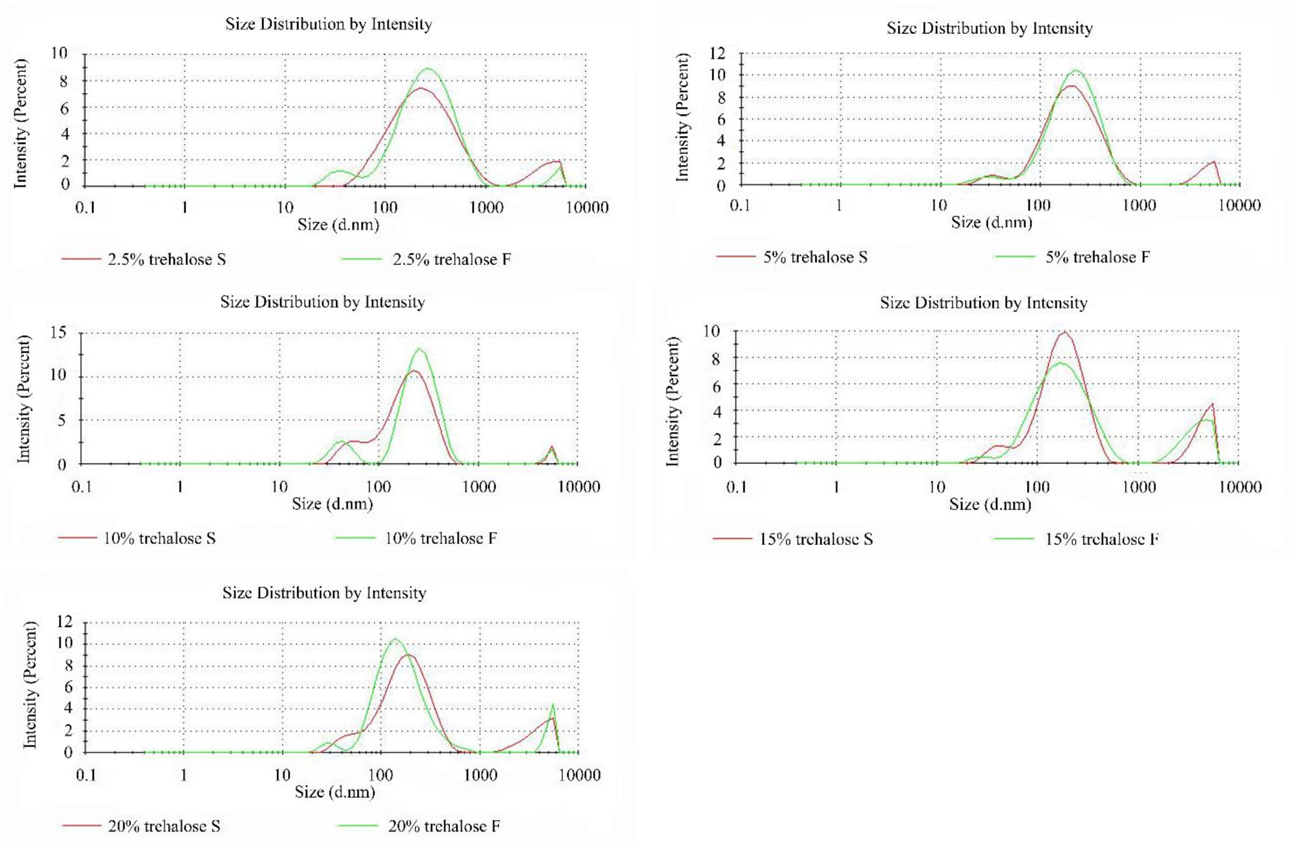

Figure S1. DLS measurements of NLC lyophilized using trehalose as cryoprotectant. S: Slow freezing speed, F: fast freezing speed.

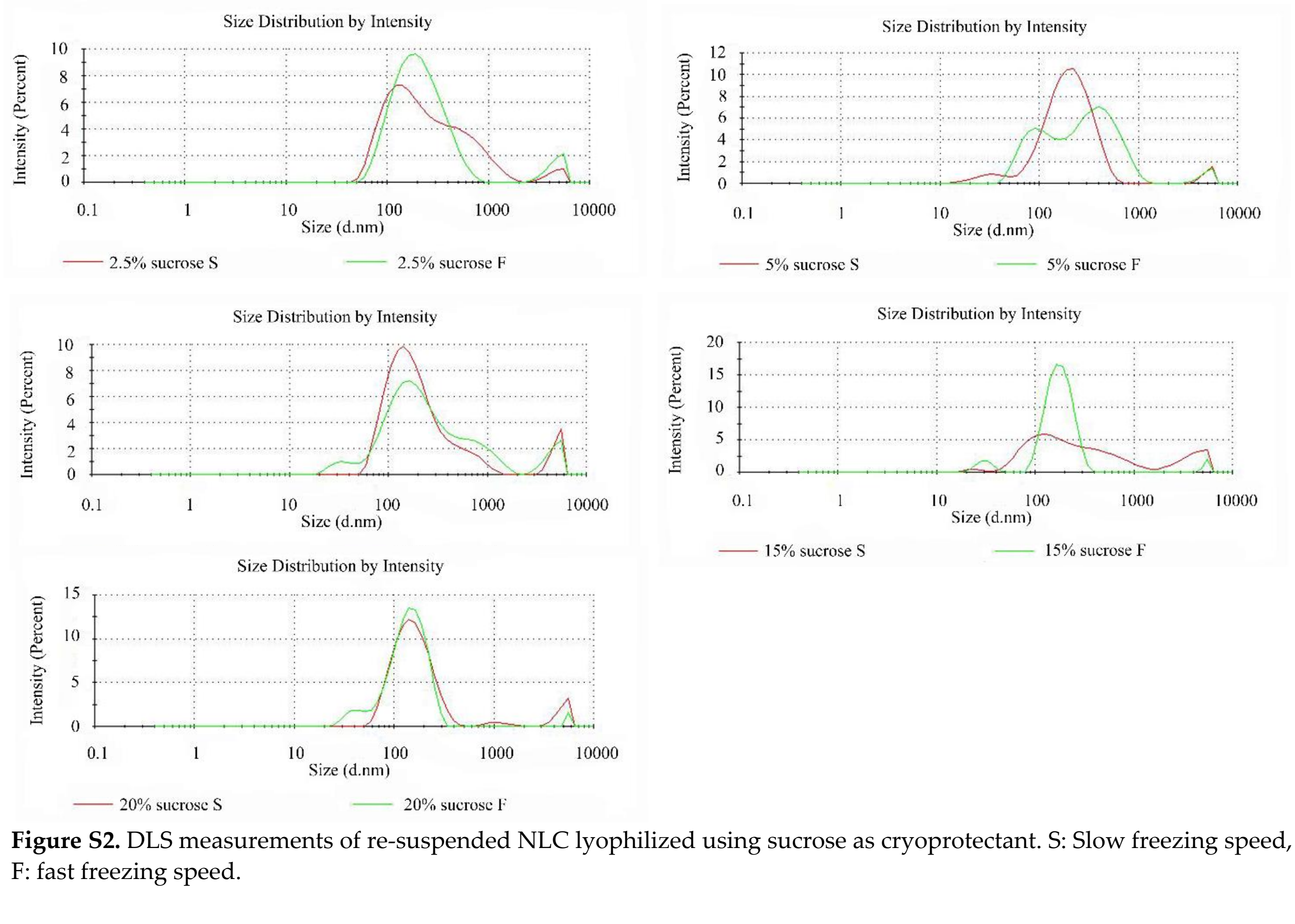

Figure S2. DLS measurements of re-suspended NLC lyophilized using sucrose as cryoprotectant. S: Slow freezing speed, F: fast freezing speed.

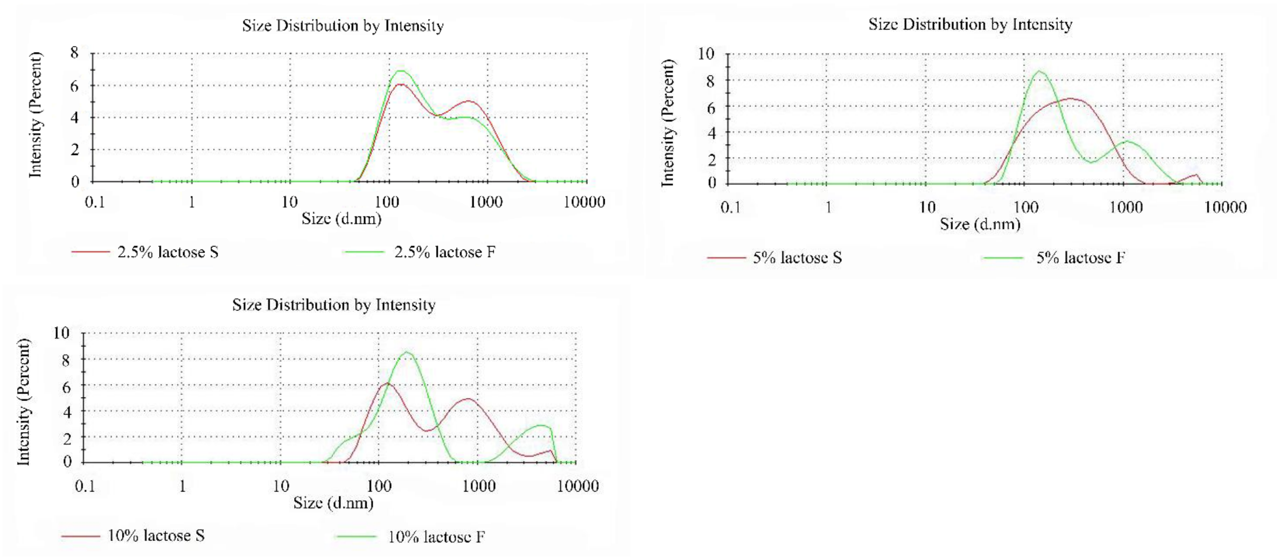

Figure S3. DLS measurements of re-suspended NLC lyophilized using lactose as cryoprotectant. S: Slow freezing speed, F: fast freezing speed.

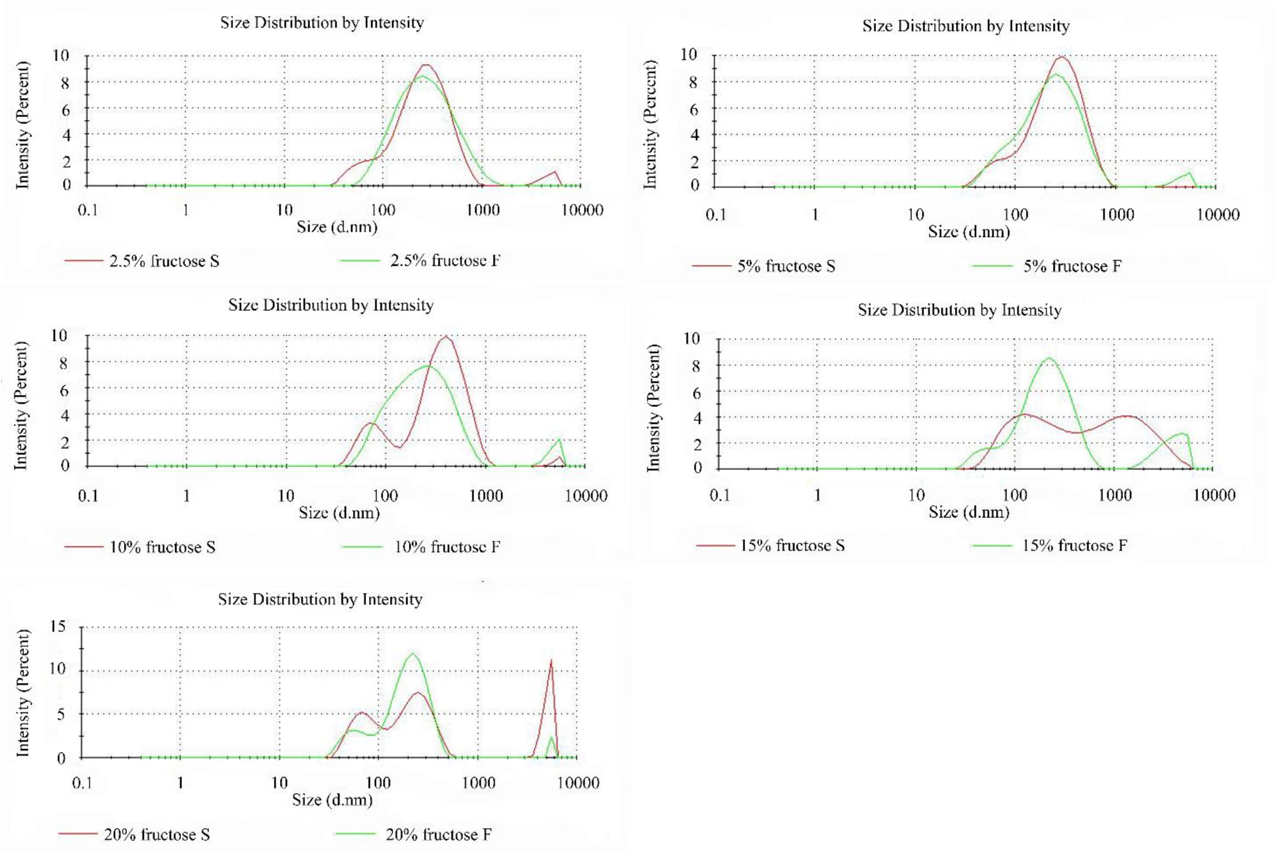

Figure S4. DLS measurements of re-suspended NLC lyophilized using fructose as cryoprotectant. S: Slow freezing speed, F: fast freezing speed.

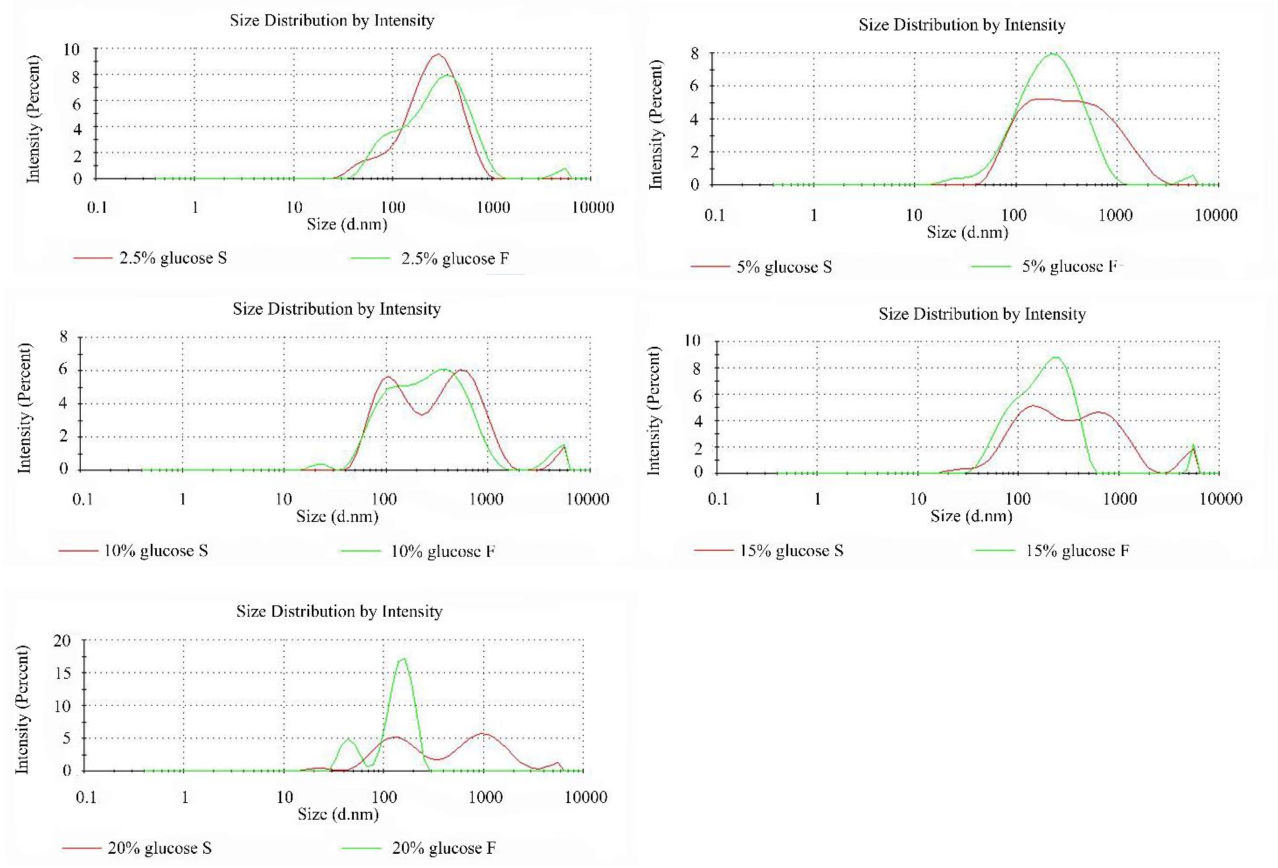

Figure S5. DLS measurements of re-suspended NLC lyophilized using glucose as cryoprotectant. S: Slow freezing speed, F: fast freezing speed.

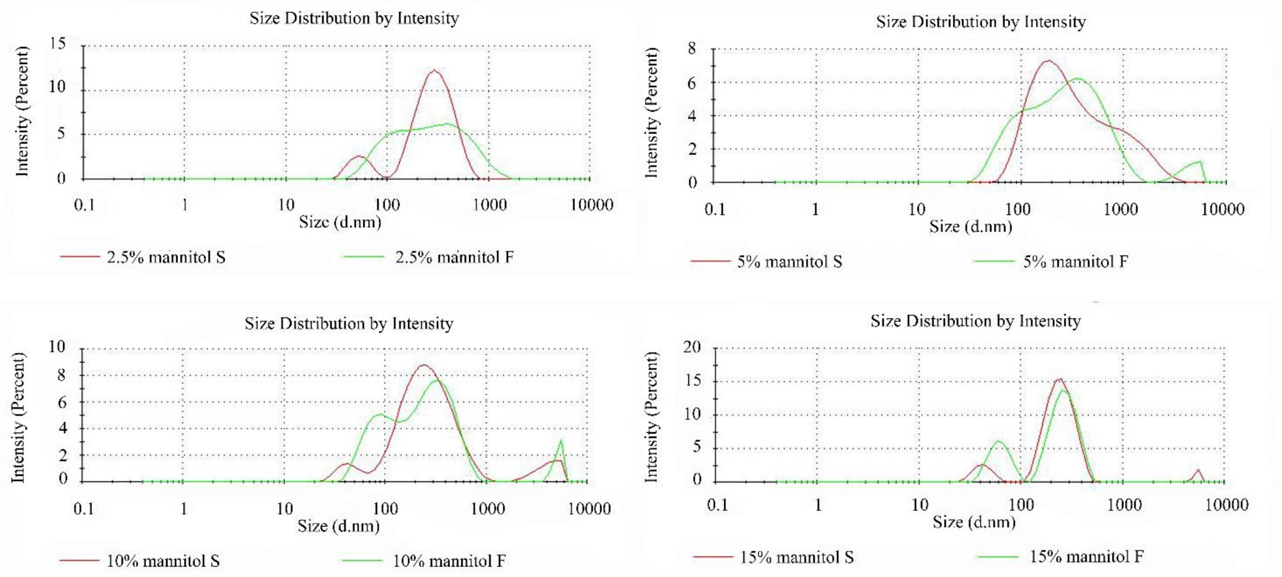

Figure S6. DLS measurements of re-suspended NLC lyophilized using mannitol as cryoprotectant. S: Slow freezing speed, F: fast freezing speed.

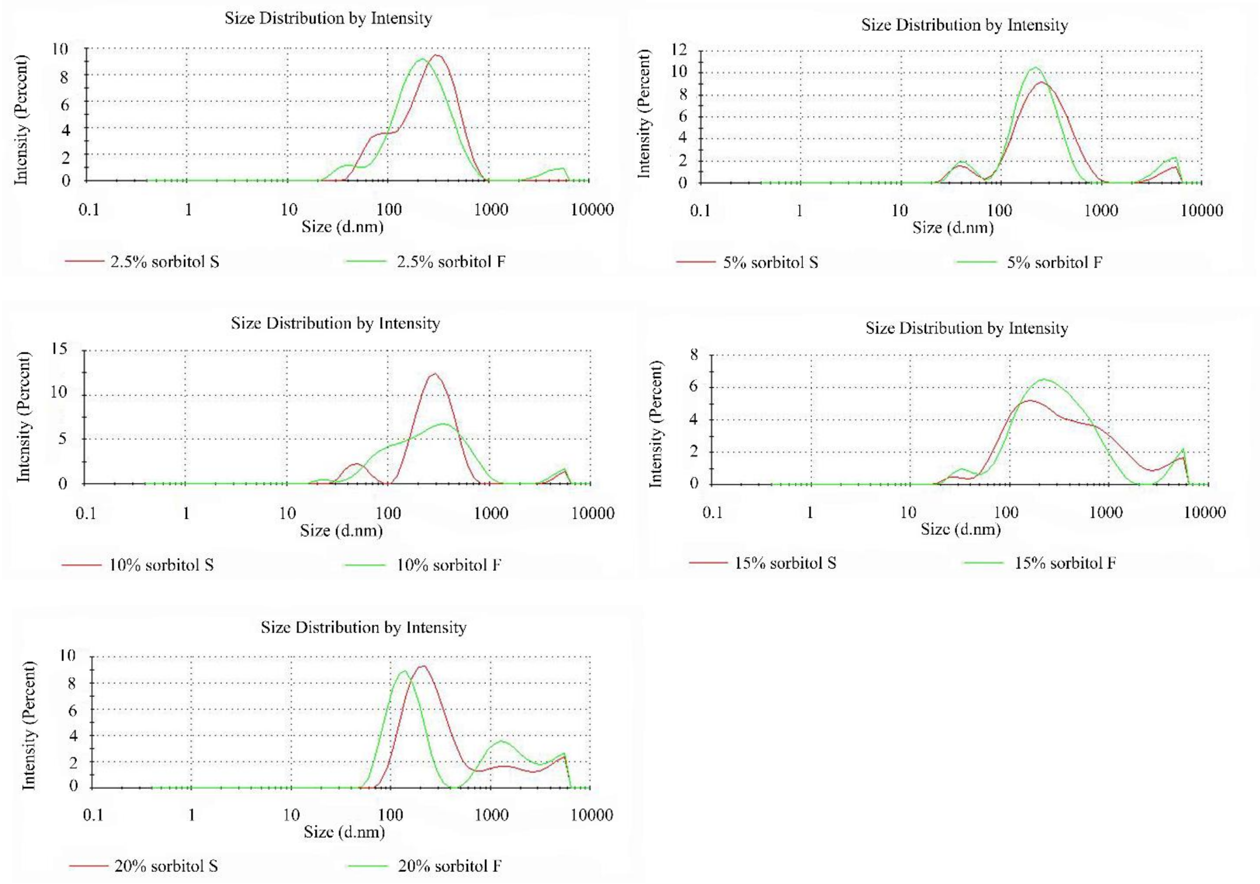

Figure S7. DLS measurements of re-suspended NLC lyophilized using sorbitol as cryoprotectant. S: Slow freezing speed, F: fast freezing speed.
